# Complete Genome of a Member of a New Bacterial Lineage in the Microgenomates Group Reveals an Unusual Nucleotide Composition Disparity Between Two Strands of DNA and Limited Metabolic Potential

**DOI:** 10.3390/microorganisms8030320

**Published:** 2020-02-25

**Authors:** Vitaly V. Kadnikov, Andrey V. Mardanov, Alexey V. Beletsky, Olga V. Karnachuk, Nikolai V. Ravin

**Affiliations:** 1 Institute of Bioengineering, Research Center of Biotechnology of the Russian Academy of Sciences, Moscow 119071, Russia; 2 Laboratory of Biochemistry and Molecular Biology, Tomsk State University, Tomsk 634050, Russia

**Keywords:** candidate phyla radiation, Microgenomates, metagenomics, complete genome, DNA replication

## Abstract

The candidate phyla radiation is a large monophyletic lineage comprising unculturable bacterial taxa with small cell and genome sizes, mostly known from genomes obtained from environmental sources without cultivation. Here, we present the closed complete genome of a member of the superphylum Microgenomates obtained from the metagenome of a deep subsurface thermal aquifer. Phylogenetic analysis indicates that the new bacterium, designated Ch65, represents a novel phylum-level lineage within the Microgenomates group, sibling to the candidate phylum Collierbacteria. The Ch65 genome has a highly unusual nucleotide composition with one strand of highly enriched in cytosine versus guanine throughout the whole length. Such nucleotide composition asymmetry, also detected in the members of Ca. Collierbacteria and Ca. Beckwithbacteria, suggests that most of the Ch65 chromosome is replicated in one direction. A genome analysis predicted that the Ch65 bacterium has fermentative metabolism and could produce acetate and lactate. It lacks respiratory capacity, as well as complete pathways for the biosynthesis of lipids, amino acids, and nucleotides. The Embden–Meyerhof glycolytic pathway and nonoxidative pentose phosphate pathway are mostly complete, although glucokinase, 6-phosphofructokinase, and transaldolase were not found. The Ch65 bacterium lacks secreted glycoside hydrolases and conventional transporters for importing sugars and amino acids. Overall, the metabolic predictions imply that Ch65 adopts the lifestyle of a symbiont/parasite, or a scavenger, obtaining resources from the lysed microbial biomass. We propose the provisional taxonomic assignment ‘*Candidatus* Chazhemtobacterium aquaticus’, genus ‘*Chazhemtobacterium*‘, family ‘*Chazhemtobacteraceae*‘ in the Microgenomates group.

## 1. Introduction

The candidate division OP11 was first described as a result of a culture-independent molecular phylogenetic survey in the sediments of the Obsidian Pool thermal spring in Yellowstone National Park (USA) [1]. The 16S ribosomal RNA (rRNA) gene sequences assigned to the OP11 group have been found in marine and freshwater sediments, geothermal pools, subsurface ecosystems, soil, and other, mostly anoxic, organic-rich environments [2]. The taxonomic status of OP11 is not clearly defined. On the basis of single-cell genome analysis, Rinke et al. [3] found that Microgenomates (OP11), Parcubacteria (OD1), and Gracilibacteria (GN02) genomes form monophyletic groups within the proposed superphylum Patescibacteria. Subsequent genomic studies revealed that Microgenomates are part of a large monophyletic group in the tree of life, the candidate phyla radiation (CPR), which almost completely lacks cultivated representatives. CPR may comprise up to 26% of total bacterial diversity, with more than 70 different phylum-level lineages [4,5,6], defined according to the levels of 16S rRNA dissimilarity. According to the NCBI taxonomy database [7], the Microgenomates group at present comprises 16 phylum-level divisions. However, the recently described Genome Taxonomy Database (GTDB) based on genome phylogeny, collapses all CPR lineages into a single candidate phylum, Patescibacteria, comprising the candidate class Microgenomatia [8].

Genomics studies of CPR bacteria [4,9,10,11,12,13,14,15,16] have revealed that they have relatively small genomes (~1 Mbp or less) and limited metabolic capacities, including the common lack of complete pathways for amino acid, nucleotide and lipid biosynthesis, that suggests a partner-dependent symbiotic or parasitic lifestyle [4,17,18]. Physical associations of CPR bacteria with their hosts have been shown for a member of Saccharibacteria (TM7), which attaches to *Actinomyces odontolyticus* [19]. Most CPR bacteria lack a respiratory chain and a complete tricarboxylic acid (TCA) cycle, consistent with a fermentation-based metabolism. However, biosynthetic capacity varies greatly across different lineages, from minimal to more complex, for example, in some members of the Parcubacteria [20].

Until now, Microgenomates have no cultured representatives, and all their genomes have been assembled from metagenomes or obtained as a result of single-cell genome sequencing. Most of these genomes are incomplete, which limits detailed analysis of the lifestyle of Microgenomates and the presence or absence of key metabolic pathways. For most lineages of Microgenomates, complete genomes are not yet available.

Previously, we studied the composition of the microbial community and sequenced the metagenome of a deep subsurface thermal aquifer located in Western Siberia, Russia [21,22]. Deep subsurface environments are extreme habitats characterised by a combination of high temperature, pressure, and salinity According to the results of 16S rRNA profiling, this microbial community consisted mainly of methanogenic archaea and various uncultured lineages of the *Firmicutes*, *Chloroflexi*, *Ignavibacteriae*, *Deltaproteobacteria,* and *Armatimonadetes* [21]. Members of the Microgenomates group were present in low numbers. 

Here, we used metagenomic data from this subsurface aquifer [22] to reconstruct the closed circular genome for a member of a new phylum-level lineage within the Microgenomates group. Genome data were used to define the phylogenetic position of the new lineage, reconstruct its metabolic pathways, and gain insights into the interactions of this bacterium with other microorganisms.

## 2. Materials and Methods

### 2.1. Site Description, Sampling, and Metagenomic DNA Isolation

The 2.8 km-deep oil-exploration borehole 5P is located in the vicinity of the Chazhemto village, Tomsk region, Russia (coordinates 58.060226 N, 82.826753 E). Samples of artesian water flowing out of the borehole were collected at the wellhead in April 2016 [22]. The water temperature was ~20 °C; it had near-neutral pH (7.43–7.6), and a negative redox potential (−304 to −338 mV). The total mineralization of the water was about 6 g/L, and its ionic content was dominated by sodium and chloride, with subsidiary calcium [22]. The water temperature was lower than expected considering a typical thermal gradient of about 20 °C per km, but it may cool when passing through the borehole.

To collect the microbial biomass, the water sample (25 L) was filtered through 0.22 μm cellulose nitrate membranes (Sartorius, Göttingen, Germany). The filters were frozen in liquid nitrogen and homogenised by grinding with a mortar and pestle. Total community DNA was extracted using a Power Soil DNA Isolation Kit (MO BIO Laboratories, Carlsbad, CA, USA). A total of about 1 μg DNA was obtained.

### 2.2. Sequencing of Metagenomic DNA Using Illumina Platform, Contig Assembly, and Binning

Sequencing of metagenomic DNA using Illumina HiSeq2500 (Illumina, San Diego, CA, USA) was described previously [22]. Sequencing of a paired-end TruSeq DNA library (2 × 250 bp) resulted in the acquisition of 57,579,354 read pairs [22]. About 16.9 Gbp was obtained upon removal of adapters, trimming low-quality sequences (Q < 33), and merging of paired reads [22]. The resulting merged and unmerged reads were de novo assembled using metaSPAdes version 3.7.1 [23].

The contigs were binned into metagenome-assembled genomes (MAGs) using the program CONCOCT [24]. Taxonomic position of assembled MAGs was determined according to the GTDB database using the GTDB-Tk v.0.1.3 tool [25]. A single MAG, designated Ch65, was assigned to the candidate order UBA1400 within the candidate class Microgenomatia of the candidate phylum Patescibacteria.

### 2.3. Sequencing of Metagenomics DNA Using the MinION System and Assembly of a Complete Genome of Ch65 Bacterium

Metagenomic DNA was additionally sequenced on MinION (Oxford Nanopore, Oxford, UK) using a Ligation Sequencing Kit 1D protocol according to the manufacturer’s recommendations. Sequencing resulted in 1,418,419 reads, with a total length of ~1.54 Gbp. 

These long reads were used to join the contigs of Ch65 MAG. For this purpose, the MinION reads exhibiting high sequence similarity to the contigs of Ch65 MAG were selected using BWA v.0.7.15 [26]. The contigs were joined by the program npScarf [27] using a SPAdes assembly graph to fill the gaps between contigs with Illumina consensus sequences (-SPAdes parameter of npScarf).

### 2.4. Genome Annotation and Analysis

A gene search and annotation were performed using the RAST server [28]. The annotation was then checked and manually corrected in a comparison of the predicted protein sequences with the National Center for Biotechnology Information (NCBI) databases. The N-terminal signal peptides were predicted by Signal P v.5.0 (http://www.cbs.dtu.dk/services/SignalP/) for Gram-positive bacteria, and the presence of transmembrane helices was predicted by TMHMM v.2.0 (http://www.cbs.dtu.dk/services/TMHMM/).

Taxonomic classification of the genomes with the GTDB was performed using the GTDB-Tk v.0.1.3 tool [25]. The average amino acid identity (AAI) between the genomes was determined using the aai.rb script from the enveomics collection [29].

The GC and AT skew plots were generated using the GenSkew online analysis tool (http://genskew.csb.univie.ac.at/).

### 2.5. Phylogenetic Analysis

The dataset used for genome-based phylogenetic analysis included Ch65 and the genomes of 44 other members of the candidate order UBA1400. For these genomes, multiple alignment of concatenated 120 bacterial single-copy marker genes was carried out using GTDB toolkit v.0.1.3. This multiple alignment, 1589 amino acids in length, was used to construct the maximum likelihood phylogenetic tree using PhyML v.3.3 [30] with the default parameters. The level of support for internal branches was assessed using the Bayesian test in PhyML.

Sequences of the 16S rRNA genes were aligned using Muscle v.3.8.31 [31]. The alignment was visualised in AliView v.1.26 [32] and introns were manually removed. The maximum likelihood phylogenetic tree was computed by PhyML v.3.3 with the default parameters. The level of support for internal branches was assessed using the Bayesian test in PhyML.

### 2.6. Nucleotide Sequence Accession Number

The annotated genome sequence of the Ch65 bacterium was submitted to the NCBI GenBank database under the accession number CP047901.

## 3. Results and Discussion

### 3.1. Assembly of the Complete Genome Sequence of Ch65 Bacterium

In order to obtain MAGs of the members of the microbial community, metagenomic sequences with a total length of about 16.9 Gbp were generated using the Illumina sequencing platforms and assembled into contigs. The binning of contigs was performed with CONCOCT [22]. One of the MAGs obtained, Ch65, consisting of six contigs, was sequenced to 66-fold average coverage. Using the GTDB-Tk v.0.1.3 tool, this MAG was identified as a member of the candidate phylum Patescibacteria.

The availability of longer reads obtained using Nanopore sequencing enabled these contigs to be joined into a single circular genome sequence. The relative abundance of this genotype in the community, defined as a fraction of Ch65 MAG in the whole metagenome, was about 0.4%.

### 3.2. General Genome Properties

The Ch65 genome is 801,504 bp long and has a G+C content of 44.80%. Single copies of the 16S, 23S, and 5S rRNA genes, and 51 transfer RNA (tRNA) genes enabling recognition of all codons for all 20 amino acids were identified. All three rRNA genes are located in one cluster also containing *trnI-GAU* and *trnA-UGC* genes between the 16S and 23S rRNA genes, but it was interrupted by an insertion of two protein-coding genes between the tRNA genes. Although the 16S rRNA gene contains no insertions, the 23S rRNA gene harbours an intron containing a LAGLIDADG-type homing endonuclease gene. The presence of introns in rRNA genes has been reported for many CPR bacteria [4].

Annotation of the genome sequence revealed 838 protein-coding genes, and functions of 403 (48%) of them were predicted. Like all CPR bacteria, Ch65 lacks genes encoding ribosomal proteins L30 and L9 [4]. The ribosomal protein biogenesis factor GTPase Der is also missing. The Ch65 genome lacks genes related to mobile elements and integrated prophages, despite the absence of a CRISPR (Clustered Regularly Interspaced Short Palindromic Repeats) system and restriction-modification enzymes that could provide protection from viruses and mobile elements. The Ch65 bacterium probably employs another mechanism for protection against invading DNA.

Analysis of the genome of Ch65 bacterium revealed no genes encoding flagellar machinery or chemotaxis. However, genes responsible for generation of type IV pili have been found. Such pili could enable twitching motility and the adhesion of the bacterium to solid surfaces [33]. Type IV pili are widespread in CPR bacteria, and it has been proposed that this system could be involved in DNA uptake [34] and in interactions with their bacterial hosts [11]. The type IV pili components are all encoded at a single locus and include major (PilA) and minor (PilE, GspJ, PilW) pilins, the prepilin signal peptidase PilD, pilus assembly ATPase PilB, retraction ATPase PilT, pilin biogenesis protein PilC, and assembly protein PilM. Notably, we did not find the *pilQ* gene for the outer membrane secretin required for export of the pilus filament across the outer membrane in Gram-negative bacteria [34]. This finding indicates that Ch65 does not have a Gram-negative cell structure. 

Ch65 cells were predicted to be rod-shaped, based on the finding of genes encoding the rod shape-determining proteins MreBCD and RodA.

Genes of the type II secretion system were found, namely, *secY*, *secD*, *secF*, *secA*, *secE,* and *secG*. The Ch65 genome also encodes a sortase, typically found in Gram-positive bacteria. Thus, the identified components provide the functions required for protein secretion, and the presence of N-terminal Sec signal peptides was detected consistently for 86 predicted proteins. Interestingly, 70 of them were annotated as hypothetical proteins with unknown functions. A total of 169 proteins were predicted to contain at least two transmembrane helices, and 23 of them also contain N-terminal secretion signals, suggesting that they could be secreted and remain linked to the cell surface. The function of only three such proteins was predicted; another 20 were annotated as hypothetical. Most of them were larger than average in size (> 300 a.a.), with the largest protein consisting of 2228 a.a. Such proteins could be involved in interactions between CPR bacteria and their hosts.

### 3.3. Phylogenetic Placement of Ch65 Bacterium

Phylogenetic analysis of Ch65 MAG by a search against GTDB [8] placed it in the candidate order UBA1400 within the class Microgenomatia. According to this classification, Ch65 was not assigned to any of the recognised candidate families within the order UBA1400, and thus, forms a novel family-level lineage in the GTDB taxonomy.

**Figure 1:**
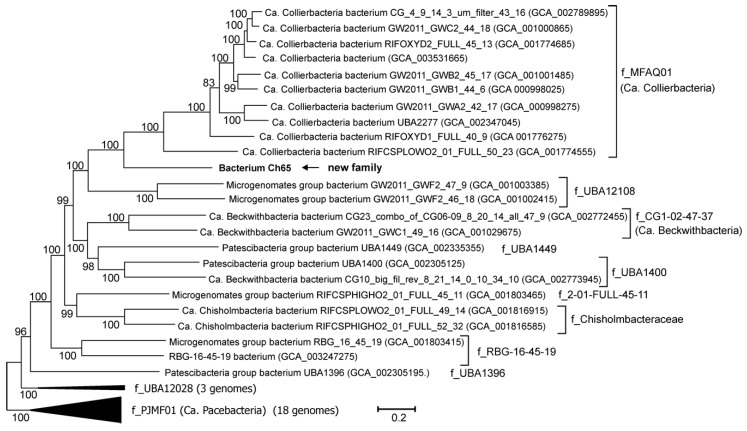
Position of Ch65 genome in the maximum likelihood concatenated protein phylogeny of the candidate order UBA1400. Taxonomy is shown according to the GTDB (f_, family), with the names of candidate phyla recognised in the NCBI taxonomy in parentheses. GenBank assembly accession numbers are shown after the genome names. The levels of support for internal branches assessed using the Bayesian test in PhyML are indicated at the nodes.

To determine the phylogenetic position of the Ch65 bacterium more precisely, a phylogenetic tree based on concatenated sequences of conservative marker genes, including Ch65 and other UBA1400 genomes, was constructed. The results confirmed that Ch65 forms a distinct family-level lineage within the candidate order UBA1400, and all families proposed by GTDB form well-separated monophyletic branches on the tree (Figure 1). Ch65 appears to form a branch sibling to the candidate family MFAQ01 (corresponding to the candidate phylum Collierbacteria in the NCBI taxonomy), and the candidate family UBA12108 forms a sister lineage.

The AAI between Ch65 and Ca. Collierbacteria (f_MFAQ01) genomes was in the range of 45.5–48.2%, and an AAI of about 43.5% was calculated for Ch65 and f_UBA12108 genomes (Table S1). The AAI between Ch65 and a member of the family CG1-02-47-37 (corresponding to the candidate phylum Beckwithbacteria in the NCBI taxonomy) was 44.3%. 

**Figure 2:**
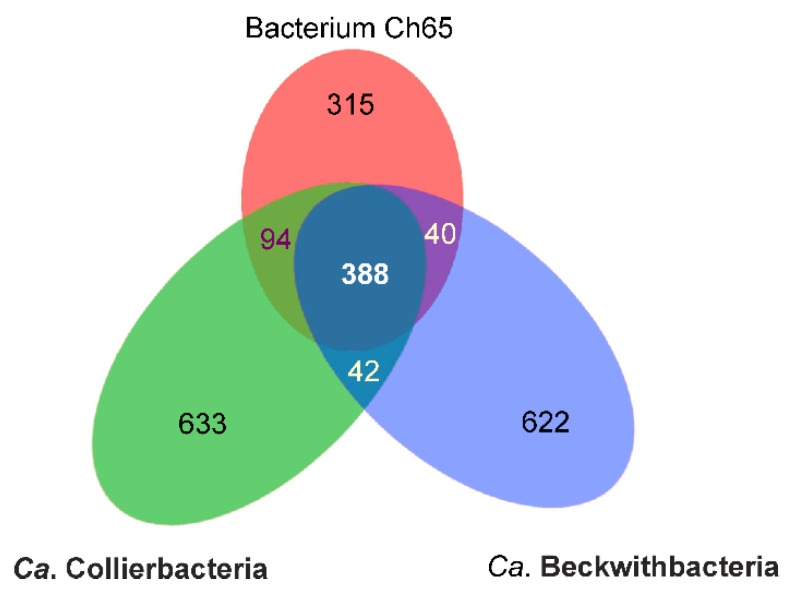
Homologous protein-coding genes of Ch65 bacterium, *Candidatus* Collierbacteria bacterium RIFOXYD2_FULL_45_13 and *Candidatus* Beckwithbacteria bacterium GW2011_GWC1_49_16. Genes were considered present in a pair of genomes if the region of similarity covered >80% of the shorter corresponding protein, with Blast *p* e-value < 1 × 10^−10^.

The availability of complete genome sequences for members of Ca. Collierbacteria (bacterium RIFOXYD2_FULL_45_13, GenBank MFAS01000001) and Ca. Beckwithbacteria (bacterium GW2011_GWC1_49_16, GenBank CP011210) allowed us to compare their predicted proteomes; 388 of the Ch65 protein-coding genes are present in all three genomes, while 95 and 40 are shared with only Ca. Collierbacteria and Ca. Beckwithbacteria, respectively; 315 genes were not found in either of them (Figure 2). Most of genes found in only one of three genomes were predicted to encode hypothetical proteins with unknown functions. Altogether, these data further support the status of Ch65 as a new family-level lineage in the GTDB taxonomy.

**Figure 3:**
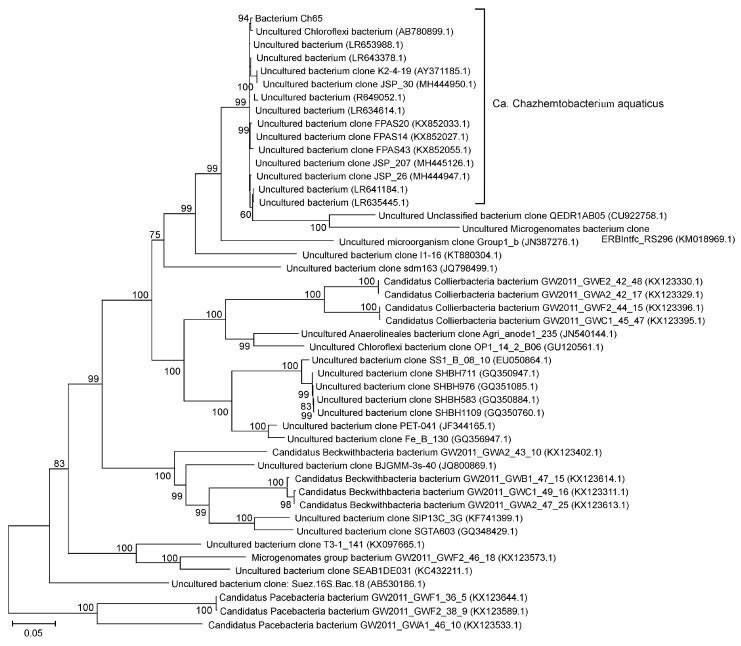
Position of Ch65 genome in the maximum likelihood 16S rRNA gene phylogenetic tree. GenBank accession numbers are shown after the clone names. The scale bar represents substitutions per nucleotide base. Bootstrap values are indicated at the nodes.

A nucleotide BLAST search against the NCBI NR database for relatives of Ch65 on the basis of 16S rRNA sequence similarity revealed 17 environmental clones that are closely related to the Ch65 bacterium, with sequence identities of more than 98%, followed by a large gap to the next more distant relative with only 89.9% sequence identity to Ch65 (Table S2). Closely related 16S rRNA gene sequences have been detected worldwide in organic-rich anaerobic environments such as anaerobic digesters, wastewater treatment plants, rhizospheric soil, and lake sediments (Table S2). On the 16S rRNA phylogenetic tree, Ch65 and related clones are clearly separated from clones representing Ca. Collierbacteria (MFAQ01) and Ca. Beckwithbacteria (CG1-02-47-37) (Figure 3). Unfortunately, the absence of near-complete 16S rRNA genes in most MAGs used to build the GTDB taxonomy and shown in Figure 1 makes it impossible to conduct a detailed comparison of genomic and 16S rRNA-based phylogenies.

The status of a lineage represented by the Ch65 genome, as well as Ca. Collierbacteria and Ca. Beckwithbacteria, as families in the candidate order UBA1400 within the class Microgenomatia of the phylum Patescibacteria, or as separate phyla within the superphylum Microgenomates, will become clear only after the acceptance of the rules for assigning species to higher taxonomic ranks and the appropriate establishment of the taxonomy of CPR lineage.

### 3.4. Nucleotide Composition Disparity

**Figure 4:**
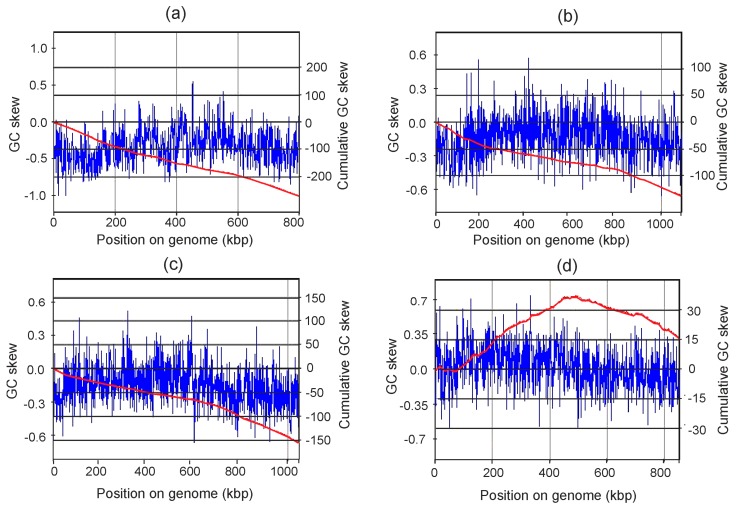
Diagram showing the GC skew (blue lines) and calculated cumulative GC skew (red lines) across the complete genomes of UBA1400 members: (**a**) Ch65 bacterium, (**b**) *Candidatus* Collierbacteria bacterium RIFOXYD2_FULL_45_13 (GenBank MFAS01000001), (**c**) *Candidatus* Beckwithbacteria bacterium GW2011_GWC1_49_16 (GenBank CP011210), (**d**) Bacterium GW2011_GWF2_28_16 of the candidate family PJMF01 (GenBank CP011212). Window size 10.000 nt, step size 1000 nt.

An interesting feature of the Ch65 genome is the unusual pattern of the cumulative GC skew calculated for the closed chromosome sequence. Asymmetries in mutational frequency, DNA repair efficiency, and a preference in the third codon position for G over C and T over A, as well as elevated levels of cytosine deamination in single-stranded DNA during replication and transcription [35,36,37], result in the majority of bacterial genomes having a leading strand rich in G and T, while the lagging strand rich in A and C [38]. The normal bidirectional replication of a bacterial genome from a single origin results in a minimum cumulative GC skew at the origin and a maximum at the terminus of replication [39]. However, GC skew profiling of the Ch65 genome revealed a continuous near-linear decrease of the cumulative GC skew across the whole chromosome length (Figure 4). No clear trend was observed for the cumulative AT skew (Figure S1). The G and C content of two strands of the genome is drastically different: the strand carrying the rRNA genes has 29.83% C and only 14.97% G, while no strong disparity was detected for A (27.87%) and T (27.33%) bases (Table 1). The G versus C content disparity is probably unrelated to gene distribution bias, since 449 and 389 protein-coding genes were predicted in C-rich and G-rich strands, respectively. Although the reasons for such G/C disparity remain to be elucidated, one could hypothesise that the origin and the terminus of replication could be located close to each other in the Ch65 genome, leading to replication of most of the chromosome proceeding in one direction. Moreover, taking into account the small genome size, a unidirectional theta-type mechanism of replication, described in some bacterial plasmids [40], could be proposed for Ch65 bacterium.

Such an unusual GC skew profile has not been reported previously for CPR genomes. For example, complete genomes of members of Ca. Gracilibacteria and Ca. Peregrinibacteria are characterised by typical cumulative GC skew curves with two clearly detectable extremes at the origin and the terminus of replication [13,16]. Therefore, we analysed the GC skew patterns for the three currently known complete genomes of other members of the candidate order UBA1400, representing Ca. Collierbacteria (GenBank MFAS01000001), Ca. Beckwithbacteria (GenBank CP011210), and the candidate family PJMF01 (GenBank CP011212). For members of Ca. Collierbacteria and Ca. Beckwithbacteria, continuously decreasing cumulative GC skew curves and a G/C content disparity between two strands similar to that found for the Ch65 genome were observed, while the UBA1364 sp001029715 genome representing the phylogenetically more distant PJMF01 family displayed a typical GC skew pattern and no G/C content disparity between two strands of the genome (Figure 4 and Table 1). Therefore, the strong disparity of G and C content between two strands of DNA and the unusual GC skew pattern could be a common property of lineages which are phylogenetically related to Ch65.

### 3.5. Predicted Central Metabolic Pathways

**Figure 5:**
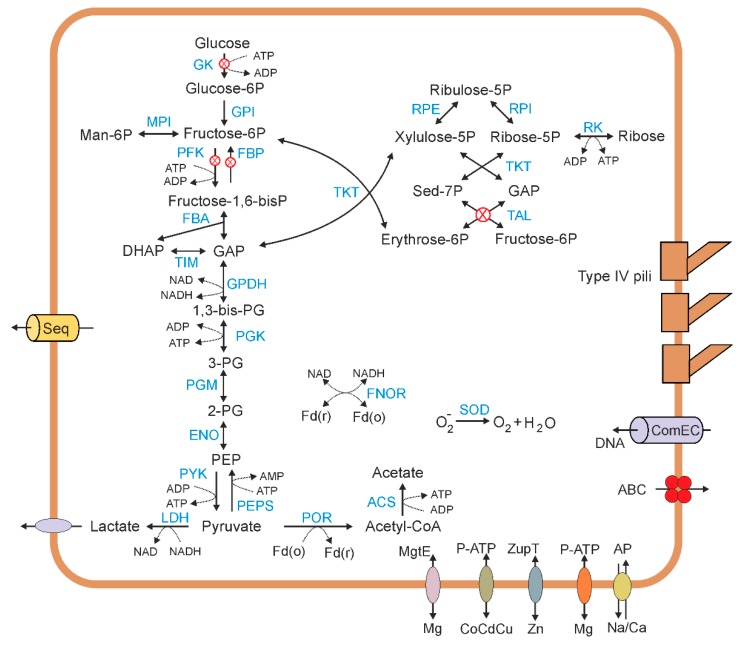
Overview of the metabolism of Ch65 bacterium. Abbreviations: GK, glukokinase; GPI, glucose-6-phosphate isomerase; MPI, mannose-6-phosphate isomerase; PFK, 6-phosphofructokinase; FBP, fructose-1,6-bisphosphatase; FBA, fructose-bisphosphate aldolase; TIM, triosephosphate isomerase; GPDH, glyceraldehyde 3-phosphate dehydrogenase; PGK, phosphoglycerate kinase; PGM, phosphoglycerate mutase; ENO, enolase; PK, pyruvate kinase; PEPS, phosphoenolpyruvate synthase; LDH, lactate dehydrogenase; POR, pyruvate ferredoxin oxidoreductase; ACS, acetyl-CoA synthetase; RPE, ribulose-phosphate 3-epimerase; RPI, ribose 5-phosphate isomerase; RK, ribokinase; TKT, transketolase; TAL, transaldolase; FNOR, ferredoxin-NADP(+) reductase; SOD, superoxide dismutase; ABC, ABC-type transporter; P-ATP, P-type ATPase; AP, antiporter; Man-6P, mannose-6-phosphate; Sed-7P, sedoheptulose-7-phosphate; GAP, glycerinaldehyde-3-phosphate; DHAP, dihydroxyacetone phosphate; 1,3-bis-PG, 1,3-biphosphoglycerate; 3-PG, 3-phosphoglycerate; 2-PG, 2-phsophoglycerate; PEP, phosphoenolpyruvate; Fd(o) and Fd(r), oxidized and reduced forms of ferredoxin. Red signs indicate missing genes from the respective pathways.

The Ch65 genome contains genes encoding most of the enzymes of the Embden–Meyerhof glycolytic pathway, including glucose-6-phosphate isomerase, class I fructose-bisphosphate aldolase, triosephosphate isomerase, NAD-dependent glyceraldehyde 3-phosphate dehydrogenase, phosphoglycerate kinase, phosphoglycerate mutase, enolase, and pyruvate kinase (Figure 5). However, the genes of two key enzymes, glucokinase and 6-phosphofructokinase, were not found. Their absence correlated with the absence of two enzymes performing the reverse reactions in the course of gluconeogenesis, fructose-1,6-bisphosphatase and glucose-6-phosphatase, while the phosphoenolpyruvate synthase gene was present. The absence of phosphofructokinase, the key enzyme linking upper and lower glycolysis, has been reported in many CPR genomes; it has been suggested that these bacteria could complete the glycolytic pathway by using a metabolic shunt, converting fructose-6-phosphate into glyceraldehyde-3-phosphate via the nonoxidative pentose phosphate pathway [9,17]. However, the nonoxidative branch of the pentose phosphate pathway in the Ch65 genome is also incomplete, since, despite the presence of genes coding for ribulose-phosphate 3-epimerase, ribose 5-phosphate isomerase, and transketolase, the transaldolase gene was not identified. Nevertheless, the Ch65 fructose-bisphosphate aldolase belongs to the class I aldolases, which also include transaldolases and fructose-6-phosphate aldolases [41]. In the Ch65 genome, this gene is located downstream of the transketolase genes in one operon. Therefore, it is possible that the transaldolase function is performed by this enzyme. Alternatively, the Ch65 bacterium could acquire intermediates of the glycolytic pathway (e.g., fructose-1,6-bisphosphate or glyceraldehyde-3-phosphate) from the environment or its host organisms. The near-complete absence of the whole glycolytic pathway in some other CPR bacteria [16,17] suggests that this could be a common strategy in this lineage.

None of the genes of the oxidative stage of the pentose phosphate pathway and the TCA cycle were identified in the Ch65 genome. Like all CPR bacteria studied to date, Ch65 lacks autotrophic carbon fixation pathways and the aerobic respiratory chain (NADH dehydrogenase, succinate dehydrogenase, complex III, and cytochrome oxidases). Terminal reductases that could perform anaerobic respiration were not found either.

The pyruvate generated during glycolysis could be decarboxylated to yield acetyl-coenzyme A (CoA) by pyruvate:ferredoxin oxidoreductase. The terminal reaction of fermentative metabolism, the oxidation of acetyl-CoA into acetate with the concomitant generation of ATP, could be catalysed by acetyl-CoA synthetase. The presence of lactate dehydrogenase suggests that lactate could also be produced as a fermentation product with concomitant re-oxidation of NADH. Reduced ferredoxin could be re-oxidised by ferredoxin-NADP(+) reductase. Hydrogenases, often used by anaerobic bacteria for the oxidation of reduced ferredoxin and NAD(P)H, coupled to hydrogen production, were not identified.

It is likely that the Ch65 bacterium produces ATP only in substrate-level phosphorylation reactions, since it lacks the genes for membrane-linked ATP synthases and any enzymes that could enable the generation of a transmembrane ion gradient.

Like most CPR bacteria studied to date [17], Ch65 lacks complete pathways for the biosynthesis of amino acids, nucleotides, and fatty acids. Therefore, all these compounds are likely to be derived from other organisms or scavenged from dead cells. In particular, the Ch65 genome encodes the DNA processing protein DprA and the competence protein ComEC, essential components of the DNA uptake machinery, and the ComEA protein involved in DNA binding [34,42]. Analysis of the Ch65 genome revealed that this bacterium, like other CPR organisms [17], has essentially complete pathways for peptidoglycan biosynthesis. Particularly notable is the presence of 29 genes of glycosyl transferases that could be involved in cell wall biosynthesis.

### 3.6. Possible Growth Substrates

Members of the CPR group have often been described as being capable of degrading complex organic substrates such as hemicelluloses [4,43,44]. Genomes of Microgenomates have been found to contain a wider inventory of glycoside hydrolases than other CPR lineages [44]. However, an analysis of the Ch65 genome revealed only five glycoside hydrolases. Among them were GH18 family peptidoglycan hydrolase, two enzymes of family GH130 that comprise phosphorylases and hydrolases for beta-mannosides, GH39 family hydrolase (known activities of GH39 are α-L-iduronidase and β-xylosidase), and alpha-amylase. Amino acid sequences of all the glycoside hydrolases lack recognisable N-terminal signal peptides, indicating the involvement of these enzymes in the intracellular metabolism of sugars, rather than extracellular hydrolysis of polymeric substrates.

A search for proteolytic enzymes revealed only four peptidases carrying N-terminal secretion signals, assigned to the C39, Do/DeqQ, S41, and S2P/M50 families. Family C39 mostly contains endopeptidases that cleave the leader peptides from the precursors of various bacteriocins [45]. Do/DeqQ and S41 peptidases, encoded at a single locus, are likely involved in the degradation of incorrectly synthesised proteins, as well as protection from thermal and other stresses. The Zn-dependent proteases of the S2P/M50 family are involved in intramembrane proteolysis for diverse signal transduction mechanisms [46]. Therefore, it is unlikely that Ch65 bacterium could rely on extracellular hydrolysis of proteinaceous substrates for growth.

Analysis of the Ch65 genome revealed the absence of a phosphotransferase system for sugar uptake, as well as ABC-type transporters for import of sugars, amino acids, and peptides. Intracellular enzymes involved in amino acid fermentation pathways (aminotransferases, glutamate dehydrogenase, etc.) were not found either. Overall, a very limited number of transporters have been identified in the Ch65 genome: sodium–calcium antiporter, magnesium-transporting P-type ATPase, ZIP family zinc transporter (ZupT), two MgtE-type Mg/Co/Ni transporters, TauE/SafE sulphite exporter, P-type heavy metal-transporting ATPase, a major facilitator superfamily transporter of unknown specificity, and three ABC-type transporters that are likely involved in the export of antimicrobial peptides. Therefore, it seems unlikely that Ch65 bacterium could import even simple sugars and amino acids/peptides via conventional transport systems. However, the Ch65 genome was predicted to encode many hypothetical membrane-associated proteins with multiple transmembrane helices that may be involved in transport functions and/or interactions with other cells. It is possible that Ch65 cells could obtain metabolites from a partner organism through cell-to-cell connections.

### 3.7. Description of the New Taxon

The genome of Ch65 bacterium meets the criteria recently suggested for description of new taxa of uncultivated microorganisms [47], and we propose the following taxonomic names for the novel genus and species of Ch65.

- Description of the novel genus *Candidatus* Chazhemtobacterium (Cha.zhem.to,bac.te’ri,um. N.L. neut. n. bacterium a rod; N.L. neut. n. Chazhemtobacterium a rod named after Chazhemto, Tomsk region, Russia).
- Description of the novel species *Candidatus* Chazhemtobacterium aquaticus (a.qua¢ti.cus L. masc. adj. aquaticus living or found in the water, aquatic).

Not cultivated. Inferred to be rod-shaped, motile, anaerobic, obligate organotroph with fermentative metabolism, lacking many pathways for biosynthesis of amino acids, nucleotides, and fatty acids. Presumably adopted the lifestyle of a scavenger or involved in symbiotic/parasitic relationships with other organisms. Represented by complete genome (acc. no. CP047901) obtained from metagenome of a deep subsurface thermal aquifer in Western Siberia, Russia. 

Based on this, we propose the name *Candidatus* Chazhemtobacteriaceae fam. nov. for the family. The family is defined on a phylogenetic basis by comparative 16S rRNA sequence analysis of *Candidatus* Chazhemtobacterium aquaticus Ch65 and uncultured representatives detected in various environments. The type genus is *Candidatus* Chazhemtobacterium. We suggest placing this family within the Microgenomates group without definition of a new order, class, or phylum until the phylogeny of CPR is established.

## 4. Conclusions

*Candidatus* Chazhemtobacterium aquaticus Ch65 represents a novel lineage of the Microgenomates group, a sibling of Ca. Collierbacteria that could be considered as a family in the GTDB taxonomy or as a novel phylum in the NCBI taxonomy. An interesting feature of the Ch65 genome is its highly unusual nucleotide composition, with one strand highly enriched in cytosine versus guanine. Such nucleotide composition asymmetry, also detected in the members of Ca. Collierbacteria and Ca. Beckwithbacteria, suggests that most or even the whole Ch65 chromosome is replicated in one direction, unlike most bacteria.

*Candidatus* Chazhemtobacterium aquaticus Ch65 is also remarkable for its unusual metabolic capabilities. It requires an external source of metabolites for the biosynthesis of lipids, amino acids, and nucleotides. The absence of some key enzymes of the glycolysis and nonoxidative pentose phosphate pathway indicates that Ch65 probably also needs to acquire the intermediates of the glycolytic pathway from external sources. The required resources could be obtained from either lysed microbial cells or from associated living hosts. Interactions of Ch65 bacterium with its hosts could be facilitated by type IV pili. Overall the metabolic predictions imply that Ch65 adopts the lifestyle of a scavenger or a symbiont/parasite, growing in microbial biofilms. The fermentation products generated by Ch65 bacterium (acetate and lactate) could support the growth of methanogens and sulphate reducers, which are abundant in the microbial community of deep subsurface aquifers.

## Figures and Tables

**Table 1:** Nucleotide composition of complete genomes of members of the candidate order UBA1400.

Lineage (GenBank acc. no)	Genome Size (bp)	GC Content (%)	Nucleotide Composition of One Strand of DNA (%)			
			G	C	A	T
Bacterium Ch65 (CP047901)	801,504	44.80	14.97	29.83	27.87	27.33
Ca. Collierbacteria (MFAS01000001)	1,089,434	44.95	19.44	25.51	27.03	28.02
Ca. Beckwithbacteria (CP011210)	1,049,888	48.92	20.66	28.26	25.33	25.75
f_PJMF01 (CP011212)	853,053	35.99	18.27	17.72	32.36	31.65

## Supplementary Materials

The following are available online at https://www.mdpi.com/2076-2607/8/3/320/s1, Figure S1: Diagram showing the AT skew and calculated cumulative AT skew across the Ch65 genome; Table S1: Average amino acid identity between Ch65 genome and phylogenetically related members of the order UBA1400; Table S2:. Environmental 16S rRNA gene sequences related to Ch65.
