# Supplementary material for: Complete Genome of a Member of a New Bacterial Lineage in the Microgenomates Group Reveals an Unusual Nucleotide Composition Disparity Between Two Strands of DNA and Limited Metabolic Potential
Source: Microorganisms. 2020 Feb 25;8(3):320. doi: 10.3390/microorganisms8030320 (PMC7143001; doi:10.3390/microorganisms8030320)
Supplement: Supplementary file 1 [file microorganisms-08-00320-s001.zip › Figure S1.pdf]

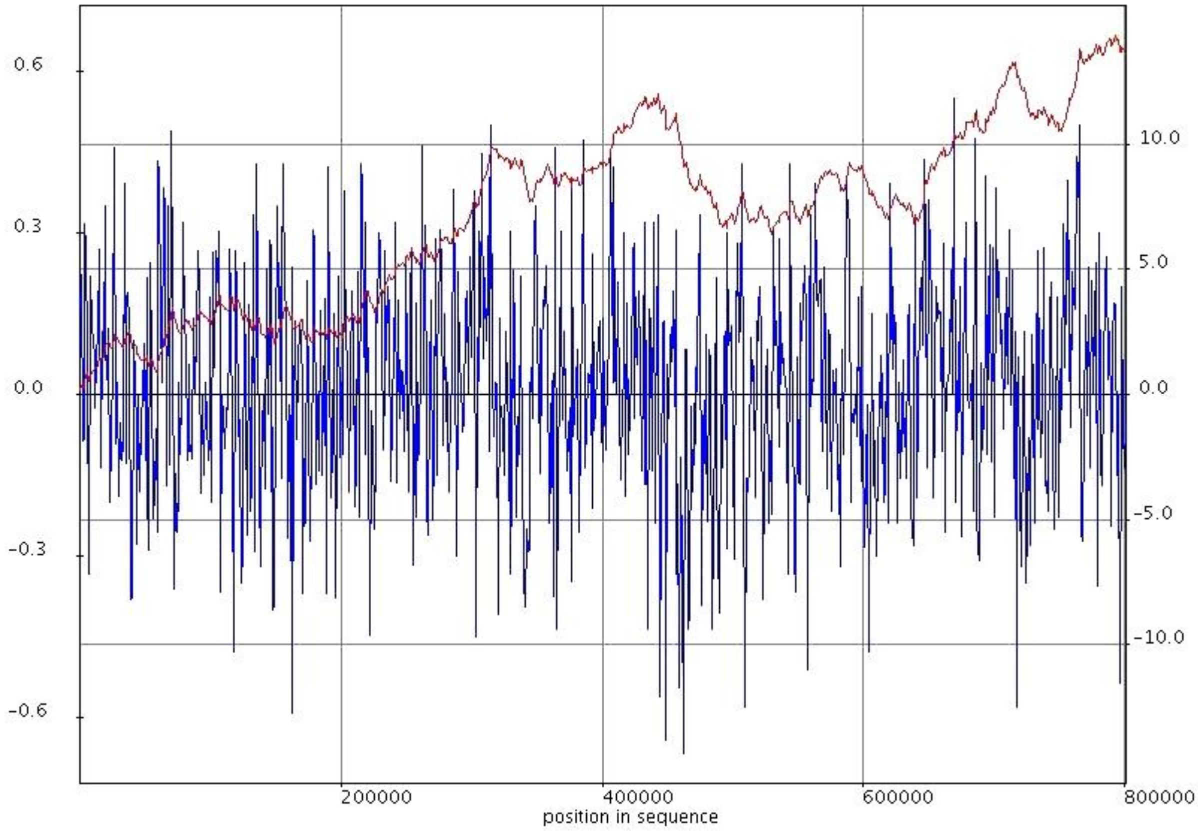

Figure S1. Diagram showing the AT skew (blue line, values are shown on the left) and calculated cumulative AT skew (red lines, values are shown on the right) across the Ch65 genome. Window size 10.000 nt, step size 1000 nt.
